# Transcriptome alterations of mitochondrial and coagulation function in schizophrenia by cortical sequencing analysis

**DOI:** 10.1186/1471-2164-15-S9-S6

**Published:** 2014-12-08

**Authors:** Kuo-Chuan Huang, Ko-Chun Yang, Han Lin, Theresa Tsun-Hui Tsao, Sheng-An Lee

**Affiliations:** 1 Department of Psychiatry, Beitou Branch, Tri-Service General Hospital, Taipei, Taiwan; 2 Department of Nursing, Ching Kuo Institute of Management and Health, Keelung, Taiwan; 3 Graduate Institute of Biomedical Electronics and Bioinformatics, National Taiwan University, Taipei, Taiwan; 4 Department of Biochemical Science and Technology, National Taiwan University, Taipei, Taiwan; 5 Department of Information Management, Kainan University, Taoyuan, Taiwan

## Abstract

**Background:**

Transcriptome sequencing of brain samples provides detailed enrichment analysis of differential expression and genetic interactions for evaluation of mitochondrial and coagulation function of schizophrenia. It is implicated that schizophrenia genetic and protein interactions may give rise to biological dysfunction of energy metabolism and hemostasis. These findings may explain the biological mechanisms responsible for negative and withdraw symptoms of schizophrenia and antipsychotic-induced venous thromboembolism.

We conducted a comparison of schizophrenic candidate genes from literature reviews and constructed the schizophrenia-mediator network (SCZMN) which consists of schizophrenic candidate genes and associated mediator genes by applying differential expression analysis to BA22 RNA-Seq brain data. The network was searched against pathway databases such as PID, Reactome, HumanCyc, and Cell-Map. The candidate complexes were identified by MCL clustering using CORUM for potential pathogenesis of schizophrenia.

**Results:**

Published BA22 RNA-Seq brain data of 9 schizophrenic patients and 9 controls samples were analyzed. The differentially expressed genes in the BA22 brain samples of schizophrenia are proposed as schizophrenia candidate marker genes (SCZCGs). The genetic interactions between mitochondrial genes and many under-expressed SCZCGs indicate the genetic predisposition of mitochondria dysfunction in schizophrenia. The biological functions of SCZCGs, as listed in the Pathway Interaction Database (PID), indicate that these genes have roles in DNA binding transcription factor, signal and cancer-related pathways, coagulation and cell cycle regulation and differentiation pathways.

In the query-query protein-protein interaction (QQPPI) network of SCZCGs, TP53, PRKACA, STAT3 and SP1 were identified as the central "hub" genes. Mitochondrial function was modulated by dopamine inhibition of respiratory complex I activity. The genetic interaction between mitochondria function and schizophrenia may be revealed by DRD2 linked to NDUFS7 through protein-protein interactions of FLNA and ARRB2.

The biological mechanism of signaling pathway of coagulation cascade was illustrated by the PPI network of the SCZCGs and the coagulation-associated genes. The relationship between antipsychotic target genes (DRD2/3 and HTR2A) and coagulation factor genes (F3, F7 and F10) appeared to cascade the following hemostatic process implicating the bottleneck of coagulation genetic network by the bridging of actin-binding protein (FLNA).

**Conclusions:**

It is implicated that the energy metabolism and hemostatic process have important roles in the pathogenesis for schizophrenia. The cross-talk of genetic interaction by these co-expressed genes and reached candidate genes may address the key network in disease pathology. The accuracy of candidate genes evaluated from different quantification tools could be improved by crosstalk analysis of overlapping genes in genetic networks.

## Background

The etiology of schizophrenia has been gaining more focus in recent brain research. One of the most intriguing areas of schizophrenia research is the identification of candidate genes from different postmortem cortical regions associated with positive and negative symptoms for the pathophysiology of schizophrenia. The neurodevelopmental studies of schizophrenia have used postmortem superior temporal gyrus (STG/BA22) tissue samples which are responsible for cognition and memory. Next generation sequencing (NGS) accelerates biological research in disease pathology such as genomics, transcriptomics, gene expression analysis[1]. Schizophrenia is a complex neurodevelopmental disorder. The vulnerability basis of schizophrenia demonstrates the genetic deficit of the complex heritability. The use of RNA-Seq technology provides a more complete dataset for transcriptome analysis than microarray technology. Six public human brain RNA-Seq datasets, as listed in Table 1, are currently available from the sequence read archive (SRA).

**Table 1:** Publicly available human brain RNA-Seq datasets on the SRA database.

Tissue location	Sample size	Disease type	Author	Organization	Publication date
temporal cortex:	6	3 Autism vs 3 Control	Xinchen Wang	University of Toronto	12-Jul-11

human reference brain RNA	16	-	Daniel Ramsköld	Karolinska Institute	16-Jul-12

caudate nucleus	5	-	Genevieve Konopka	UT Southwestern Medical Center	9-Nov-11

frontal pole	6	-	Genevieve Konopka	UT Southwestern Medical Center	9-Nov-11

hippocampus	6	-	Genevieve Konopka	UT Southwestern Medical Center	9-Nov-11

Superior temporal gyrus	18	9 schizophrenia vs 9 control	Jing Qin Wu	The Ramaciotti Centre, NSW, Australia	9-May-12

Accumulating evidence suggests that mitochondria dysfunction is one of the pathological mechanisms for schizophrenia. Genetic variations in mitochondrial DNA polymorphism and antipsychotic-induced weight gain are associated with schizophrenic subjects[2]. The ATP level was decreased in the left temporal in schizophrenic patients[3] and mitochondrial DNA common deletion in brain samples and polymorphisms are associated with schizophrenic patients[4,5], suggesting that the alteration of mitochondria and dysregulation of energy metabolism may contribute to implication of schizophrenia[6,7].

Venous thromboembolic events have been associated with psychosis in unmedicated schizophrenic patients[8]. Evidence indicates that abnormal tissue plasminogen activator (tPA) activity is an important predisposing factor for schizophrenia[9]. Moreover, chronic anticoagulation therapy is associated with remission of psychotic symptoms, which suggest that imbalance of tPA levels in the brain may affect the stabilization of psychotic symptoms[10]. Proteomic study provided evidence that serum abnormalities in schizophrenic patients involved in phosphorylation of proteins in coagulation pathways[11]. It has been suggested that drug-induced sedation, obesity and enhanced platelet aggregation result in increased activity in the coagulation system[12-14].

The studies by Huang et al.[15], Wu et al.[16] and Sellmann et al.[17] used human postmortem brain tissue samples for schizophrenia research. The previous post-mortem brain studies in schizophrenia by Huang et al.(2013) contains 19 control and 23 schizophrenia microarray samples of BA22 brain tissue[18]. Sellmann et al.(2013) used 10 schizophrenia patients and 10 controls in their microarray study. KJ Brennand et al.(2011) collects 4 samples from schizophrenia patients and 4 controls to derive the human induced pluripotent stem cells. There are few RNA-Seq datasets available in post-mortem brain samples mostly because of its high cost. RNA-Seq is a high-throughput sequencing technique as an alternative to microarray for genotyping, which its sequencing data are highly replicable with little technical variation, and it enables more accurate identification of differentially expressed genes, alternative splice variants and novel transcripts[19]. The SRA database contained 36 public NGS biosamples associated with schizophrenia. Therefore, the 9 schizophrenic samples and 9 controls collected by Wu et al. used in this study are of reasonable sample size for transcriptome analysis. The human brain area STG/BA22 is believed to be associated with speech and language, known as Wernicke's area, and responsible for many positive symptoms and cognitive dysfunction involving auditory processing and social cognition in schizophrenia. Wu et al. revealed three functional clusters highly relevant to schizophrenia in STG/BA22 with the use of RNA-Seq[16]. Using systems biology and bioinformatic analysis tools, the pathology and disease mechanisms for schizophrenia were gradually resolved to discover new biological pathways, targets and new treatment strategy. Sun J. et al.(2010) reported schizophrenic networks and pathways[20]. Disease networks were drawn for this complex disease[21], and the relationship between schizophrenia and cancer has also been investigated[15].

In Phenopedia, the most reported schizophrenia-related gene was COMT, which was supported by 278 publications; whereas DRD2 and BDNF are supported by 139 and 116 publications respectively. ATF3 is consistently under-expressed and appears in four different databases in both RNA-Seq and microarray analyzed brain samples, further evidence suggest that the ATF3 associated TREM-1 gene has significantly increased expression in monocytes of schizophrenia patients[22]. The association of schizophrenia and the under-expression of the FOXN3 gene has been reported[23,24].

A complementary research to evaluate the deficit in schizophrenia requires GWAS analysis of candidate genes, SNP changes of specific gene, clinical quantitative endophenotypes, genetic network and protein functionality from microarray, and NGS data of peripheral blood and postmortem brain samples. This study explores the SCZCGs consistently found in different tissue-specific datasets, focusing on the genotypes of hemostasis and energy metabolism in schizophrenia. To illustrate the pathology of schizophrenia, differentially expressed candidate genes were constructed into protein-protein interaction (PPI) networks. Relevant pathways and protein complexes were analyzed to discover novel cellular function or disease mechanism by systems biology in schizophrenia. Moreover, the co-expressed SCZGCs from different schizophrenic datasets were used to demonstrate the relationships of coagulation and mitochondrial function in schizophrenia.

## Methods

### Selection of differential expression genes by quantifying transcript abundances of postmortem brain tissue in schizophrenia

The raw reads datasets (SRA: ERP001304, BioProject: PRJEB2939) were downloaded from the NCBI SRA database. In ERP001304[16], total RNA was extracted from postmortem BA22 STG tissue from 9 schizophrenic patients and 9 matched controls sourced from the NSW Tissue Resource Center, the University of Sydney, Australia. Other publicly available human brain RNA-Seq datasets in NCBI SRA database are listed in Additional file 1.

We aligned the FASTQ data of ERP001304 to the reference human transcriptome using Bowtie 1 and sorted using SAMtools[25]. In order to quantify transcript abundances, RSEM[26] was used to normalize RNA-Seq data for each sample into transcripts per million (TPM). The significant differentially expressed transcripts with over- or under-expressed genes in the BA22 STG brain specimen were selected by using the Student's t-test between the schizophrenia and control samples. The corresponding t-test of each gene with a p-value less than 0.05 was defined as candidate genes for schizophrenia (SCZCGs).

### Analysis flow of NGS datasets related with postmortem brain tissue in schizophrenia

**Figure 1:**
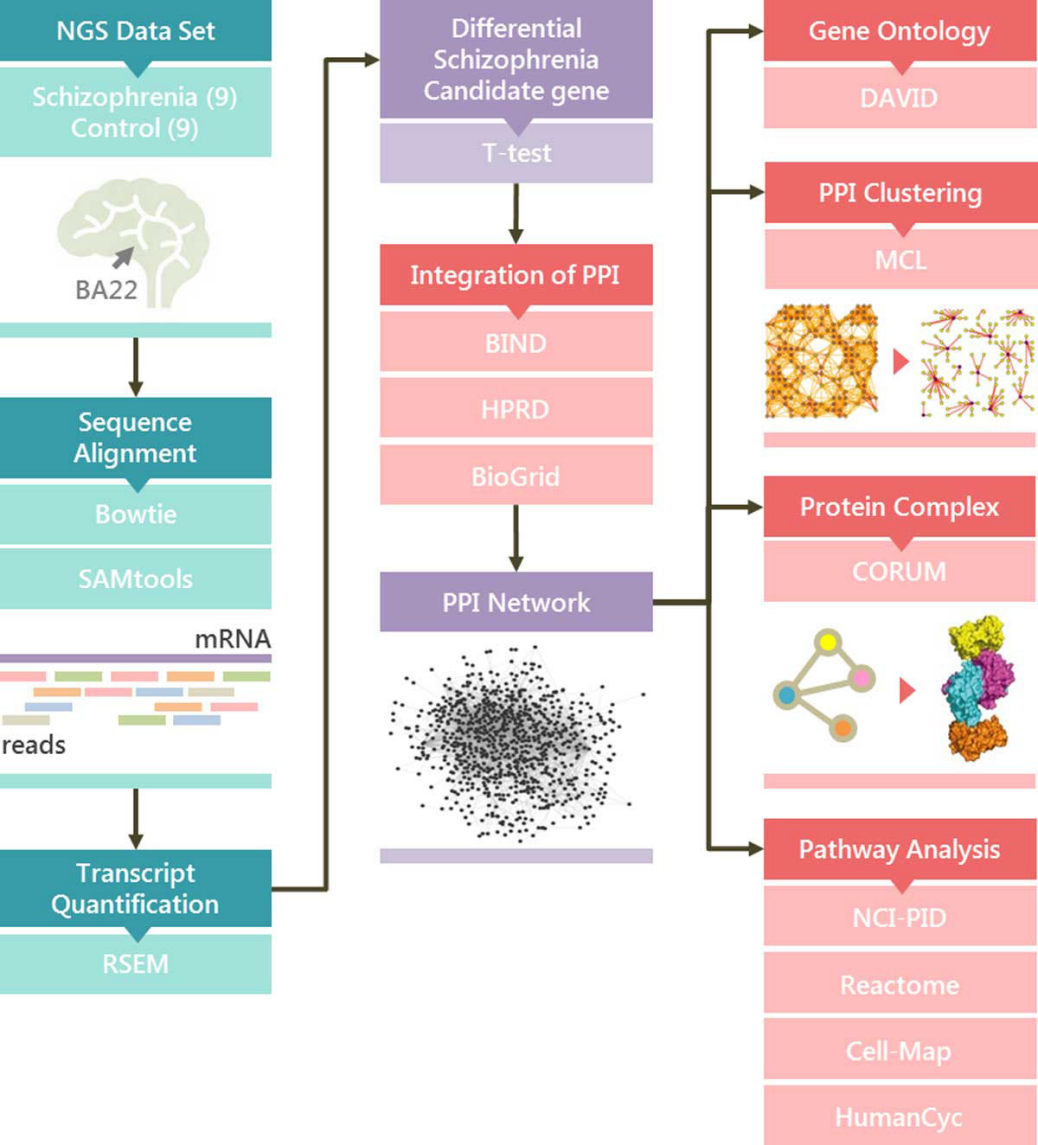
**The analytical flow discovering candidate genes and pathways**. By analysis of NGS data from BA22 STG sample, the significant differential genes were screened by Bowtie 1 and RSEM to construct the QQPPI network for schizophrenia. The potential complexes or pathways which were searched against pathway and protein complex databases such as NCI-PID, Reactome, Cell-Map, HumanCyc and CORUM are obtained by MCL cluster with the corresponding bioinformatic tools. The right part shows the corresponding analytical tools for the analysis of candidate PPI subnetworks and pathways.

A key challenge in transcript quantification from RNA-Seq data is the handling of reads that map to multiple genes or isoforms. We used tools such as Bowtie 1[27] and Tophat[28] to align the reads. We also used quantification tools such as Cufflinks[29] and RSEM[26] to estimate the abundance and differential expression of transcripts. Different quantification measures used in transcriptome analysis could result in different sets of candidate genes[30]. RSEM computes the maximum likelihood abundance using the Expectation-Maximization (EM) algorithm for optimal distribution of multiple hit reads[31]. The primary output of RSEM is a measure of abundance in terms of transcripts per million (TPM). The TPM measure is preferred over the RPKM and FPKM measures because it is independent of the mean expressed transcript length and is thus more comparable across samples[30]. The analytical process for the discovery of candidate genes, protein-protein interactions and corresponding pathways are illustrated in Figure 1. Student's t-test was performed to identify significant differentially expressed genes as SCZCGs.

To illustrate the pathology of schizophrenia, differentially expressed candidate genes, protein-protein interactions (PPIs), related pathways and protein complexes were analyzed to discover novel cellular functions or disease mechanism by systemic biology in schizophrenia[15,20]. The PPIs were collected from BIND[32], HPRD[33] and BioGrid[34] to construct the schizophrenic PPI network.

### Analysis of schizophrenic mediator network

With the SCZCGs, we constructed the schizophrenic candidate genes and mediator network (SCZMN) to illustrate the PPI network of SCZCGs with mediators. Mediators were defined as protein nodes which interact with SCZCGs and have a degree greater than one (i.e. have interaction with proteins in addition to the SCZCGs).

The mediator genes could manifest to the characteristic of SCZMN in disease pathways. The SCZMN was searched against DAVID[35], MCL[36] and CORUM[37] for biological functions, subnetwork clustering and potential protein complexes.

### Schizophrenic network analysis with fold-change subnetwork clustering

By applying the Markov cluster algorithm (MCL)[36,38], the combination of corresponding modulation from each node was calculated. Each node in SCZMN was weighted by fold change (FC) which was calculated by dividing the schizophrenic expression mean (SM) by the control expression mean (CM). To account for the co-regulatory relationship between two nodes, FC of adjacent nodes were multiplied to represent the edge relationship of two neighbouring nodes and used as the MCL input. The co-regulation of subnetworks in schizophrenia was analyzed by MCL to explore the biological components and processes in schizophrenia.

### Analysis of regulatory relationship between pathways involving schizophrenic candidate genes

Pathway analysis is the building process of identifying protein interactions, associated annotation and domain knowledgebase[39]. The pathway enrichment analysis was performed with PID[40], Reactome[41], Cell-Map[42] and HumanCyc[43] databases to obtain the potential pathways for the pathophysiology of schizophrenia.

For obtaining potentially involved pathways in schizophrenia, the pathway enrichment analysis of the significant differentially expressed genes are prioritized and the significance of corresponding pathways is ranked by p-value with FDR less than 0.05 with the Benjamini-Hochberg procedure using fdrtool[44].

## Results

### Exploration of schizophrenia candidate genes from different literature datasets

The significantly differential expressed genes were selected based on the curated database of literature reviews, laboratory reports, microarray differential expression analysis, generic risk prediction and RNA-Seq data analysis. Several novel candidate genes and potential pathways were associated to the susceptibility mechanism of schizophrenia. The candidate genes reported by SZGene[45], Huang et al.(2013)[15], Sellmann et al.(2013)[17], Ayalew M et al.(2012)[46], Wu et al.(2012)[16] and SCZCGs were compared and revealed high inconsistency of putative genes and pathways, results are listed in Additional file 2. NCBI ClinVar[47] provides pathogenic, protective and risk factors associated with schizophrenia, which was used to investigate the relationship between clinical manifestation and candidate genes.

The phenomenon of inconsistency of reported schizophrenic candidate genes are common between 9 different studies. Only 15 schizophrenic candidate genes with count numbers above 4 have large numbers of publications. The less candidate genes been mentioned in studies (counts), the less publication reports support. There are 3688 schizophrenic candidate genes mention only once in the 9 studies. 620 of them have been reported in literature including NRG1 which has 107 publication reports mostly in GWAS and animal study but not been reported in human post-mortem studies. Even the candidate genes in human induced pluripotent stem cell (HiPSC) study are greatly different from human brain studies, the inconsistent result may result from different tissue samples, measurement techniques, quantification analysis tools and experimental condition. It is implicated the characteristic of heterogeneity and diverse transcriptome expression in schizophrenia.

Many identified cellular functions and proteins as well as candidate genes are involved in schizophrenia. There are total 3505 schizophrenic candidate genes reported in literature. The studies of Huang et al., Wu et al. and Sellmann et al. used human postmortem brain tissue sample, there are also studies using other tissue-specific specimens including human blood and cell culture experiments from schizophrenic patients. The DISC1 and BDNF genes are the top most reported schizophrenic candidate genes. The second most reported candidate genes are MTHFR, GAD1, COMT, ATF3, and APOE, which are reported in at least four literature reviews. The results reported by different databases are highly inconsistent with different papers reporting mostly different candidate genes.

### Schizophrenic candidate genes by different alignment and quantification tools

A key challenge in transcript quantification from RNA-Seq data is the handling of reads that map to multiple genes or isoforms. RSEM computes maximum likelihood abundance estimates using the Expectation-Maximization (EM) algorithm for its statistical model[31]. The primary output of RSEM is a measure of abundance in terms of transcripts per million (TPM). The TPM measure is preferred over the popular RPKM and FPKM measures because it is independent of the mean expressed transcript length and is thus more comparable across samples[30].

Wu et al. used the TopHat and Cufflinks pipeline for alignment and tests for differential expression. In this study, the same RNA-Seq data was analyzed by Bowtie 1 for alignment and RSEM for transcript quantification to obtain candidate genes (Additional file 3). There are 722, 511 and 961 candidate genes reported by Wu et al., SCZCGs and SZGene, respectively. Only the genes FGF1 and SPARCL1 appear in all three datasets. 62 identical candidate genes appear in both SZGene and Wu et al. 31 identical candidate genes exist in both SZGene and SCZCGs. There are 30 candidate genes in both Wu et al. and SCZCGs. There are total 369 under-expressed genes and 353 over-expressed candidate genes in schizophrenia from Wu et al. There are 23 under-expressed and 6 over-expressed candidate genes (S100A13, RPS14, RPRM, NUDT3, MAP4K4 and FGF1) identical to SCZCGs. It was implicated that different analytical alignment and quantification tools may result in partially similar but complementary candidate gene groups. Only 2 genes appear in all three datasets: FGF1 and SPARCL1. SNPs in FGF1 were reported to be associated with the risk of developing schizophrenia[48]. Wu et al. reported 11 under-expressed genes and 2 over-expressed candidate genes in the mitochondria. However, there is only one candidate gene (NDUFB11) involved in mitochondrial function in SCZCGs.

**Figure 2:**
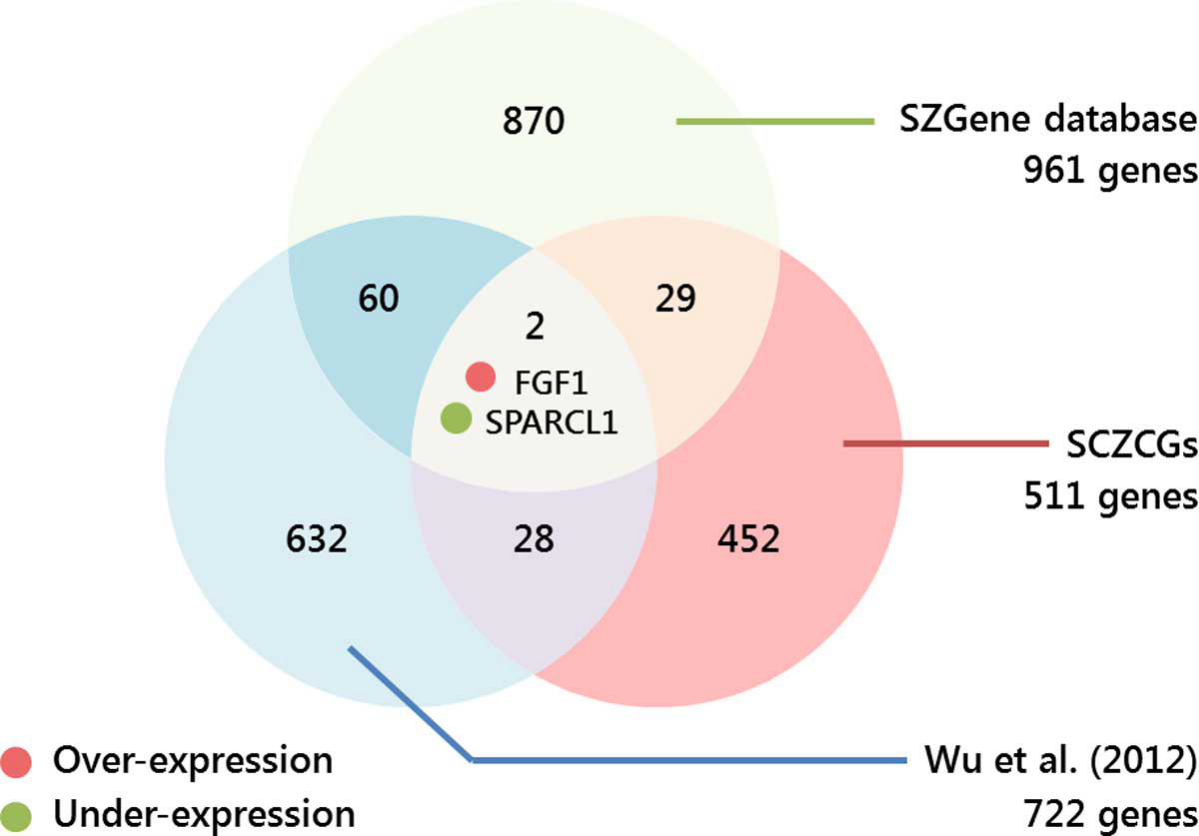
**Venn diagram of overlapped candidate genes in NGS BA22 brain samples and SZGene**. This diagram shows the heterogeneous variation of result with different quantitative tools. Candidate genes from Wu et al.(2012) were selected by TopHat+Cufflinks, whereas SCZCGs were selected by Bowtie 1+RSEM. There are 30 candidate genes appeared in both Wu et al. and SCZCGs. Only 2 candidate genes existed simultaneously in SZGene and NGS BA22 brain samples.

In Figure 2, Wu at al. reported 722 candidate genes using the Tophat and Cufflinks pipeline. However, using the same RNA-Seq data with Bowtie 1 and RSEM resulted in 511 candidate genes (SCZCGs). These results show that the use of different alignment and quantification tools may result in a completely different set of candidate genes with little consistency, and even more so when compared with the 781 candidate genes in SZGene. 62 genes appear in both Wu el al. and SZGene, 31 genes appear in both SCZCGs and SZGene. Total 91 genes were selected from Wu et al. and SCZCGs which were matched in SZGene. These matched genes may indicate the more robust candidate genes for schizophrenia with publication supports. However, there are 28 overlapped genes appear in both candidate genes of SCZCGs and Wu et al. but not in SZGene, which they may be implicated the potential candidate genes for schizophrenia and need validation in the future study.

The few overlapped candidate genes selected from different quantification tools and measurement conditions may reflect the heterogeneity of complex disease such as schizophrenia. It may implicate the importance of mediator genes as the key role of disease susceptibility which is more conserved in the candidate genetic network of schizophrenia[15]. Similarly, the inconsistency of schizophrenic candidate genes was also showed by Wu et al. and Huang et al. using the same RNA-Seq datasets of post-mortem brain sample by different quantification tools. There are 30 overlapped candidate genes from 722 and 511 candidate genes by Wu et al. and Huang et al. The 30 candidate genes may represent the co-expressed genetic interactions by different quantification measurement. The cross-talk of genetic interaction by these co-expressed genes and reached candidate genes may address the key network in disease pathology. The 30 candidate genes contains 4 PPIs which was formulated by 7 candidate genes including SLC25A5, PRKACA, APEX1, NUDT3, RPS14, FGF1 and S100A13. There are 7 candidate genes formulated 94 PPIs which contain 73 candidate genes from both Wu et al. and Huang et al. If queried 1233 candidate genes from Wu et al. and Huang et al., there are 41 candidate genes in top 63 candidate genes (count ≥ 3), the overlapped rate is 3.3%. However, there are 12 genes found in top 63 candidate genes from 73 candidate genes in cross-talk genetic interaction of Wu et al. and Huang et al. The overlapped rate improved up to 16.4% with 5 times increase. The accuracy of candidate genes evaluated from different quantification tools could be improved by crosstalk analysis of overlapped genes in genetic networks.

The genes reported by both Wu et al. and SZGene are involved in biological functions such as mitochondrial ATP synthase complex, platelet degranulation, beta-amyloid binding protein, DNA binding, transcription activity, calcium binding protein and cell cycle. However, the biological functions of genes reported by both Huang et al. and SZGene are calcium ion binding, cell cycle, platelet degranulation, MAPK activity, DNA binding, glycogen debranching enzyme activity, ATP binding, fatty acid metabolic process, cytoskeleton structure, transcription activity and ubiquitin ligase complex. The genes reported in multiple datasets have similar biological function, and results in higher confidence of potential candidate genes for schizophrenia. It also indicates that the molecular network and biological processes are likely to be disturbed in schizophrenia patients[49]. Differential expressed gene exposure to atypical antipsychotic quetiapine are cell cycle-associated in the frontal cortex[50].

### Query-Query PPI (QQPPI) network analysis of over- and under- expressed candidate genes in schizophrenia

**Figure 3:**
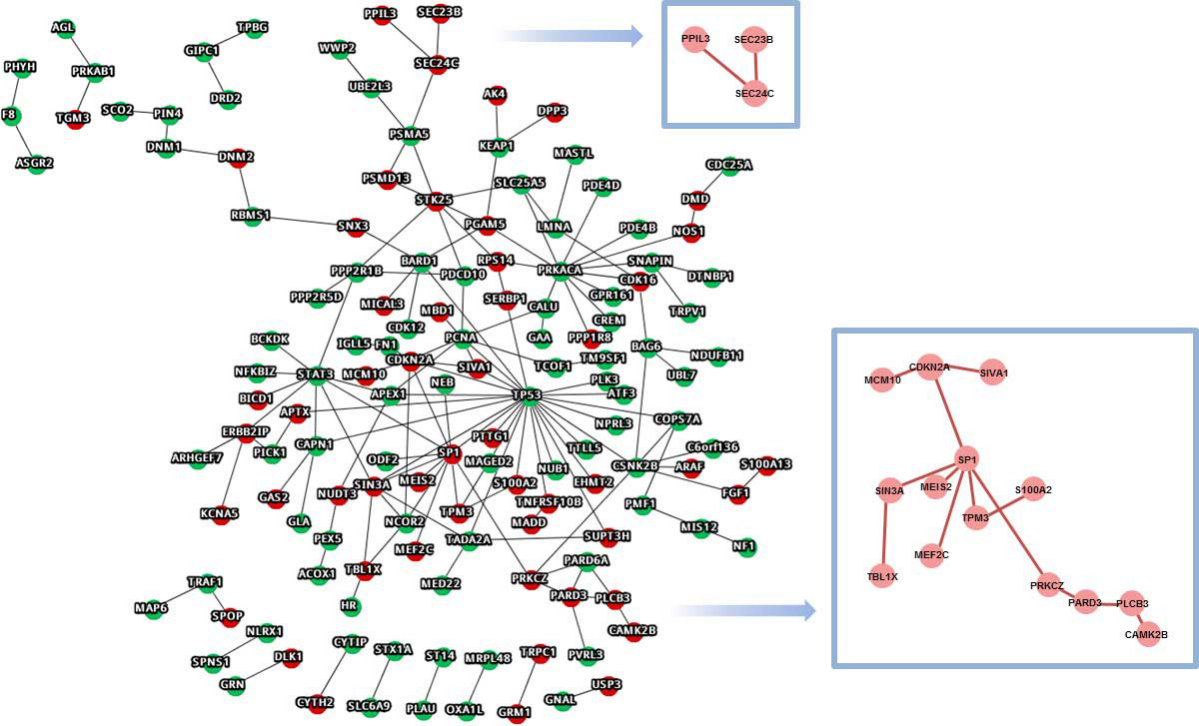
**BA22 NGS differential expression query genes in QQPPI network**. The QQPPI network formulated by SCZCGs illustrates the comprehensive genetic interactions for schizophrenia. Two subnetworks with continuous linkage of over-expressed candidate genes might reveal the potential pathways and biological function for pathophysiology of schizophrenia.

A total of 511 candidate genes in SCZCGs including 160 over-expressed and 351 under-expressed genes are used to construct a QQPPI network of schizophrenia[51]. The network consists of 156 genes with 184 PPIs as illustrated in Figure 3. The zoomed areas show the linked over-expressed subsets. PRKACA, TP53, SP1 and STAT3 have the highest degree centrality, in which STAT3, TP53 and PRKACA are under-expressed genes, while SP1 is an over-expressed gene. The SEC24C gene is associated with neurotransmitter transporters[52], while FGF1 and S100 family are related to schizophrenia[48,53].

The maximal subnetwork of 14 over-expressed genes includes MCM10, CDKN2A, SIVA1, TBL1X, SIN3A, SP1, MEF2C, MEIS2, TPM3, S100A2, PRKCZ, PARD3, PLCB3 and CAMK2B. In this subnetwork, SP1 has been reported to be abnormally expressed in schizophrenia[54]. SP1-dependent abnormal expression results in dysfunction of the mitochondrial complex I subunit[55]. SIN3A and TPM3 have also been reported to be associated with schizophrenia[56,57]. MEF2C is associated with adaptive selective pressure and the potential role in learning and memory[58]. The maximal subnetwork of over-expressed genes was searched against CORUM, in which the PLCB3-PARD3-PARD6A complex[59] was found. The complex induces transcriptional activation in intracellular Ca^2+ ^and the Wnt signaling pathway. The Wnt pathway is crucial for synaptic plasticity and its defect may contribute to the pathogenesis of schizophrenia[60,61]. It is implicated that PLCB3-PARD3-PARD6A complex may be associated with the pathology for schizophrenia. Other schizophrenia-associated complexes in SCZCGs are also searched against CORUM which are listed in Table 2. The complexes involve spliceosome, mitochondrial ribosome and other proteins which mediated regulation transcriptions, second-messenger-mediated signaling, ribosome biogenesis and protein transport.

**Table 2:** SCZCGs associated with CORUM complexes and its GO characterization.

Complex name in CORUM	SCZCGs (^:over-expressed gene, *:under-expressed gene)	GO characterization
Spliceosome	LSM4*, CDK12*, PPIL3^, THOC5*, EFTUD2 *	RNA splicing and binding

55S ribosome, mitochondria	MRPL52*, MRPL15*, MRPL48*, MRPL43*	Protein biosynthesis, mitochondria matrix

39S ribosomal subunit, mitochondria	MRPL52*, MRPL15*, MRPL48*, MRPL43*	Protein biosynthesis, mitochondria matrix

VEGF transcriptional complex	APEX1*, CITED1*, STAT3*	Regulation of transcription

PLCB3-PARD3-PARD6A complex	PLCB3^, PARD3^, PARD6A*	Regulation of transcription, second messenger-mediated signaling

Nop56p associated pre-rRNA complex	SLC25A5*, RPS14^, TCOF1*	Ribosome biogenesis

AP3-BLOC1 complex	AP3S2*, SNAP1N*, DTNBP1*	Protein targeting and transport

### Schizophrenic candidate pathway analysis with FDR adjustment

**Figure 4:**
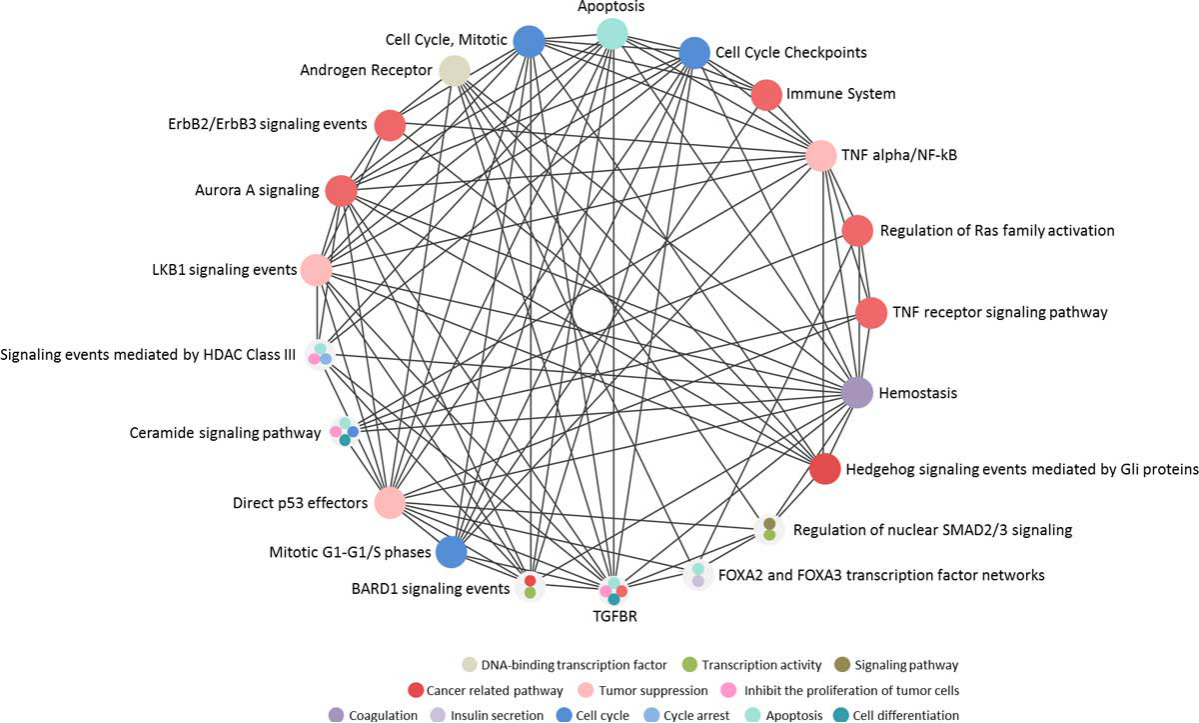
**The crosstalk of PID candidate pathways from over- and under-expressed candidate genes for schizophrenia**. The enrichment pathways with over- and under-expressed candidate genes indicated the pathway involving biological function including transcription activity, signaling pathway, cancer-related pathway, tumor suppression, coagulation, insulin secretion, cell cycle, cell differentiation and apoptosis.

The pathway enrichment analysis was performed on SCZCGs to select candidate pathways in schizophrenia using multiple pathway databases including PID, Cell-Map, Reactome and HumanCyc. 624 enrichment pathways are derived from SCZCGs in which 112 pathways were selected by their significance in p-value and FDR using the Benjamini-Hochberg procedure (Additional file 4). Alteration of DARPP-32 expression level in STG of postmortem brain was found in schizophrenia[62], which highlight the importance of pathological change of dopamine and glutamate systems in schizophrenia. In order to explore the interactions and biological functions among those enrichment pathways, the enrichment pathways from PID were analyzed for crosstalk activity (Figure 4). The interacting pathways involving biological function may also contribute to the pathology of schizophrenia which includes transcription activity, signaling pathway, cancer-related pathway, tumor suppression, coagulation, insulin secretion, cell cycle, cell differentiation and apoptosis.

Pathways reported to be associated with pathogenesis of schizophrenia include apoptosis[63], immune system[64], TNF signaling pathways[65], hemostasis[66], p53 pathway[67], BARD1 signaling pathway[68], ceramide signaling pathway[69], ErbB2 signaling pathway[70] and androgen receptor pathway[71] and HDAC signaling pathway[72,73].

### Differential gene expression and mediator network clustering analysis

Although the SCZCGs represent the significant differentially expressed genes of schizophrenia, they may not be sufficient to present the whole picture of schizophrenia. Many mediator genes may be involved in the genetic interactions without being detected as they are not usually in abundance. In order to further analyze the disease mechanism for schizophrenia, the SCZMN was constructed using the genetic interactions of SCZCGs and mediator genes.

The SCZMN that consisted of 5361 PPIs may potentially represent the most comprehensive schizophrenia-associated genetic network. The SCZMN was decomposed into clusters for analysis using the MCL algorithm. The MCL algorithm is an efficient graph clustering algorithm which helps classify SCZMN into differential expression groups of optimal subnetworks. The algorithm partitions SCZMN into clusters that are measured by edge weight of the corresponding nodes. Each cluster was calculated according to their p-value with corresponding expressed genes and mediators. The involved over- and under-expressed genes in MCL clusters with p-value are listed in Additional file 5.

In cluster 1, one of the highly ranked complexes searched against CORUM is the USP1-UAF1 complex which was reported as a schizophrenic candidate complex, and its inhibitor, Pimozide, is one of the antipsychotics with the neuroleptic property of treating schizophrenic patients[74]. It has been postulated that the USP1-UAF1 complex is associated with pathogenesis for schizophrenia. The SIRT1 gene is another candidate gene in cluster 1, which was associated with schizophrenia in a haplotype-wise analysis and may play an important role in the pathophysiology of schizophrenia in the Japanese population[75].

Cluster 39 contains under-expressed genes such as DRD2, KIF21A and PTPRU. It also contains GRIA2, SNPs within GRIA2 has been report to influence the response to antipsychotic treatment in schizophrenia[76]. However, the new antipsychotic such as paliperidone has changed the serum level of coagulation factors VIII and IX in rats[77]. One of the mediator genes of cluster 39, NCS-1 gene can mediate desensitization of D2 dopamine receptors[78]. The NCS-1 protein expression was decreased in the prefrontal cortex and in T lymphocytes and NK cells of schizophrenic patients[79]. Besides, SLC6A3 polymorphisms are associated with schizophrenia[80].

The complexes searched against CORUM revealed that the 2AR-mGluR2 complex involved in altered cortical process might be potential target for treatment of schizophrenia due to interaction with HTR2A (2AR) and metabotropic glutamate receptors (mGluR)[81]. It is implicated that there are potential complexes in cluster 39 which may be involved in the pathogenesis of schizophrenia. The functional annotation of MCL in SCZCGs, searched against DAVID with cluster number over 30, was shown in Table 3.

**Table 3:** Functional annotation of MCL in SCZCGs by DAVID.

Cluster No.(cluster genes >30) (SCZCGs/cluster genes)	SCZCGs in gene modules by MCL (^:over-expressed gene, *:under-expressed gene)	Functional annotations by DAVID
QUERY Cluster: 1 (21/215)	APEX1*,C1orf86*,DHRS7B*, DTNBP1*,EPO*,GFRA2*, KATNAL1^,NPRL3*,NUB1*, PCNA*,PLK3*,SHKBP1*,SMG9*, SPNS1*,STRADA*,SUPT3H^, TP53*,TREX1*,TTLL5*,VPS52*, ZNF174*	Regulation of transcription, DNA-dependent Regulation of RNA metabolic process

QUERY Cluster: 2 (16/154)	CAMTA2*,CDC42EP2*,FBXL19*,FOXN3*,KRT81*,MEF2C^,MPST*,MYOCD*,MYPOP*,NCOR2*, NFKBIZ*,PLA2G4C*,SIN3A^, SPARCL1*,SQRDL*,ZNF200*	Transcription regulator activity DNA binding Phosphoprotein

QUERY Cluster: 3 (6/83)	ADCY10^,GOLGA7*,LMNA*, MASTL*,ODF2*,PCNXL3*	Cytoskeleton Spliceosome Methylation Telomere maintenance ATP binding Regulation of apoptosis MAPK signaling pathway ErbB signaling pathway Cell cycle Actin filament binding Protein kinase activity

QUERY Cluster: 7 (3/40)	C16orf59*,PRKCZ^,TRIM4^	Protein kinase cascade ATP binding Tyrosine-protein kinase Phosphate metabolic process

QUERY Cluster: 9 (3/31)	LMO3^,MCM10^,SDCBP2*	DNA replication Cell cycle ATP binding

QUERY Cluster: 10 (3/31)	FN1*,IGLL5*,ST14*	Hemostasis

### Mitochondrial dysfunction in schizophrenic candidate genes

**Figure 5:**
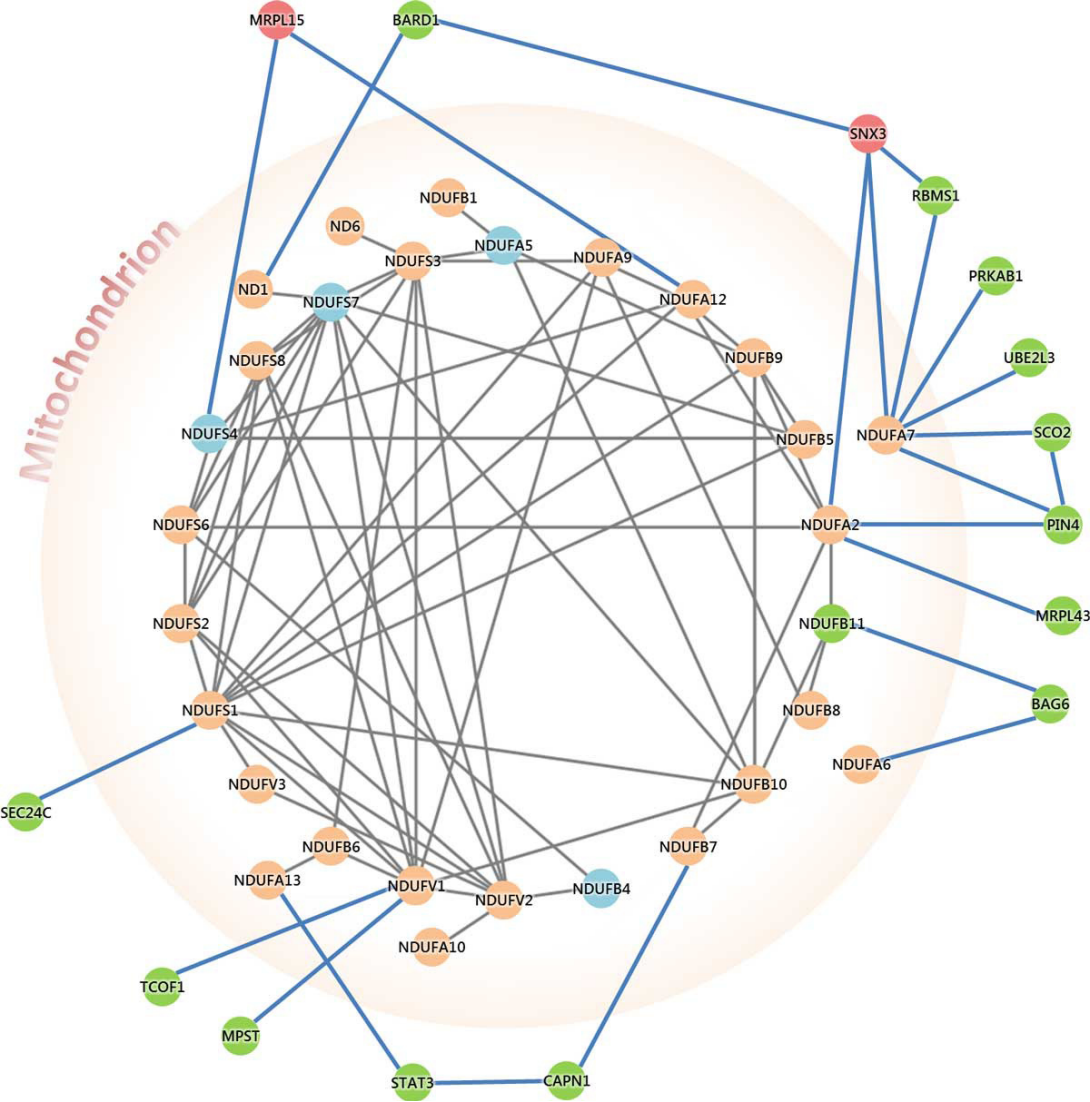
**Genetic interactions for mitochondria dysfunction in schizophrenic candidate genes**. This figure illustrates the genetic interaction network for mitochondrial associated genes and schizophrenic candidate genes. The blue nodes located inside the mitochondria denoted the under-expressed schizophrenic candidate genes from Wu et al. Red and green nodes located outside from mitochondria(except NDUFB11) represent over and under-expressed candidate genes from this study. NDUFB11 was found in both candidate genes from Wu et al. and this study. It indicates the mitochondrial associated under-expressed candidate genes manifested by mitochondrial dysfunction may contribute to the susceptibility of negative symptom for schizophrenia.

Dopamine modulates mitochondrial function through inhibition of respiratory complex I activity by abnormal interaction between dopamine and mitochondrial function[82]. The genetic interaction for mitochondria function and schizophrenia were illustrated by DRD2 linked to NDUFS7 through protein-protein interactions of FLNA and ARRB2[83,84]. Treatment with psychotropics might ultimately enhance energy metabolism and reduce the damage of oxidative stress[85]. SNX3 and MRPL15 are over-expressed candidate genes which interact with mitochondrial functions. BARD1, RBMS1, PRKAB1, UBE2L3, SCO2, PIN4, MRPL43, BAG6, NDUFB11, CAPN1, STAT3, MPST, TCOF1 and SEC24C are all under-expressed genes which interact with the Respiratory chain complex I in mitochondria (Figure 5).

Wu et al. listed 4 candidate genes (NDUFB4, NDUFS4, NDUFA5 and NDUFS7) located in the inner membrane of mitochondria, however, in this study 15 candidate genes are found (except NDUFB11) not located in the mitochondria but interact and regulate mitochondria functions. They may turn off or disturb the biological function of mitochondria and energy metabolism in schizophrenia.

Wu et al. found 17 major candidate genes associated with the Mitochondrial Complex I Subunits that contains 44 mitochondria genes according to CORUM. Since the mitochondria is responsible for vital biological processes such as energy metabolism, calcium buffering and apoptosis, it indicates the importance of mitochondrial dysfunction in the manifestation of schizophrenia[6].

### Schizophrenic differential gene expression in coagulation

**Figure 6:**
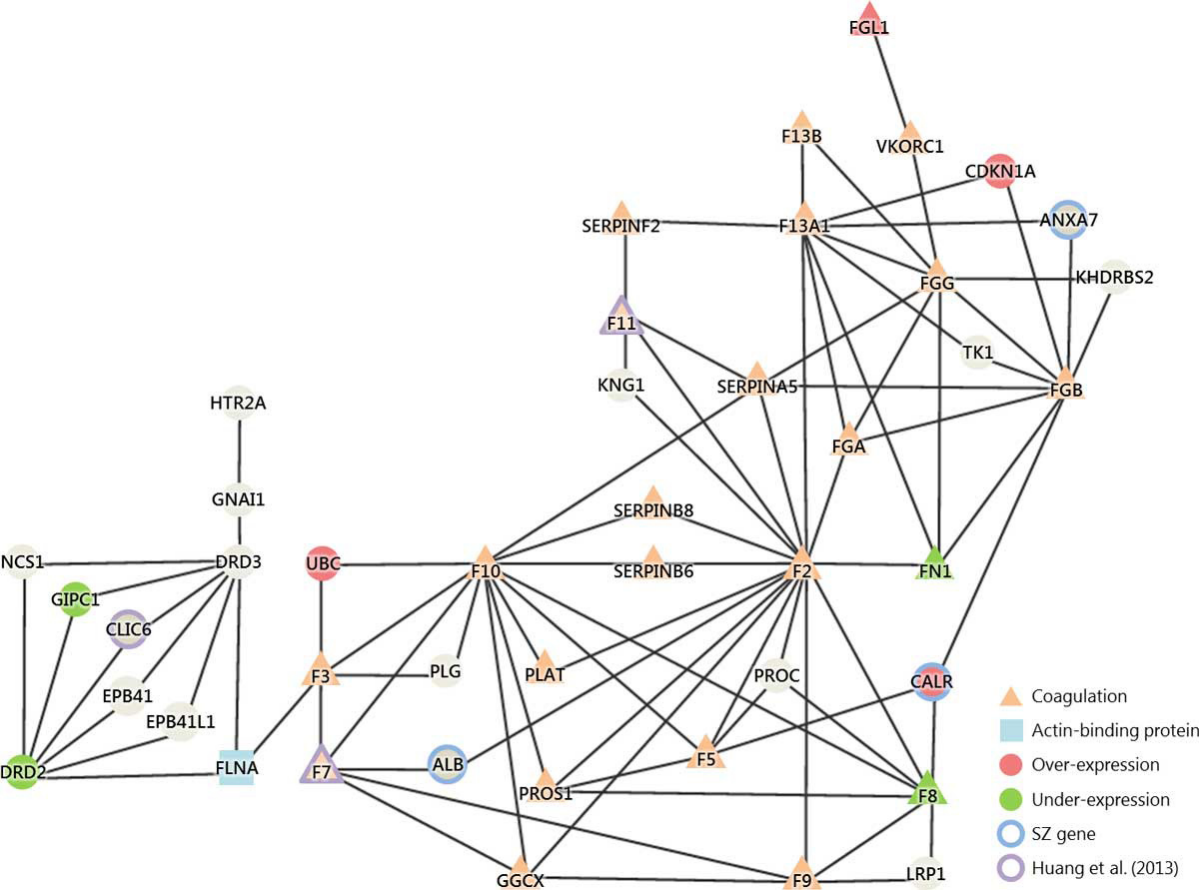
**Genetic network of coagulation function in schizophrenia**. The genetic network of hemostatic process implicates the interactions between antipsychotic target genes such as DRD2/3 and HTR2A and coagulation factor genes such as F7, F3, and F10 through bridging of actin-binding protein(FLNA) which implicates the bottleneck of this network to cascade the following coagulation function with different candidate genes in schizophrenia.

The candidate genes for schizophrenia are compared with datasets of different brain samples and literature databases. To investigate the relationship between hemostasis and schizophrenia, the hemostatic PPI network of differentially expressed candidate genes was analyzed and illustrated in Figure 6 . Huang et al. reported 12 differentially expressed candidate genes in the hemostasis pathway of the Reactome database which consists of F8, FN1, SIN3A, NOS1, OLR1, PLAU, PRKACA, PRKCZ, RAD51B, TP53, CALU and PICK1. Similarly, Wu et al. reported 18 candidate genes in the hemostasis pathway of the Reactome database which consists of HMG20B, GP1BB, HRAS, HIST2H3A, KCNMB1, ATP2B2, PPIA, PRKACA, PRKCG, KLC2, SOD1, TBXA2R, TF, TUBA4A, MAFK, CALM2, CALM3 and CD63.

Figure 6 illustrates gene interactions from different schizophrenic candidate gene datasets, such as under-expressed genes (DRD2, F8, FN1 and GIPC1) and over-expressed candidate genes (CALR, CDKN1A, FGL1 and UBC). There are many coagulation related mediator genes in the schizophrenia-coagulation gene interaction network such as F2, F3, F5, F7, F8, F9, F10, F11, F13A1, F13B, FGA, FGB, FGG, FN1, GGCX, PLAT, PROS1, SERPINA5, SERPINB6, SERPINB8 and SERPINF2. Two of them (FN1 and F8) are under-expressed coagulation genes in SCZCGs.

Many genes in the network have been reported in previous studies. ALB, ANXA7 and CALR are candidate genes in SZGene. The under-expressed candidate genes reported by Huang et al.(2013) are F11, F7 and CLIC. The over-expressed candidate gene (FGL1) and under-expressed candidate gene (CDKN1A) have been reported by Sellmann et al.(2013). HTR2A and DRD3 are listed as risk candidate genes for schizophrenia in ClinVar. HTR2A, DRD2 and DRD3 interact with the coagulation gene F3 through FLNA which causes a critical bottleneck and cascade into the following coagulation pathways. It may be implicated as the potential hemostatic mechanism for antipsychotic-induced thromboembolism.

## Discussion

### Mitochondria and schizophrenia

The mitochondrial dysfunction may decrease brain pH by magnetic resonance spectroscopy studies in living patients[86]. Mitochondrial dysfunction such as the accumulation of mitochondrial DNA damage and reactive oxygen species (ROS) production have underlined the pathological mechanism for schizophrenia[87]. Reduced numbers of mitochondria in striatum and impaired mitochondria activity in the caudate nucleus have been associated with increased risk of schizophrenia[88]. Antioxidants seemed to improve mitochondrial performance. Niacin, one of the antioxidant, could improve the manifestation of schizophrenic symptoms. NNMT, HCAR2 and QPRT are targets of nicotinic receptors. Reduced NNMT mRNA level was noted in post-mortem schizophrenic patients[89]. QPRT-involved kynurenine pathway of cortical metabolism is impaired in schizophrenia[90]. HCAR2 protein was significantly decreased in schizophrenic group[91]. Decreased niacin receptor responses were manifested in schizophrenia, and niacinamide is neuroprotective which is potentially associated with the pathogenesis of schizophrenia[92]. Treatment of niacin could improve mutism in a patient with mitochondrial encephalopathy and schizophrenia[93].

### The potential regulatory pathway for hemostasis in schizophrenia

It has been postulated that hemostatic markers of thrombogenesis are increased in acute psychotic patients. Increased serum level of coagulation factor VIII and platelets are reported in acute psychotic patients[94]. Thromboembolism in schizophrenic patients may result from pathological change of schizophrenic candidate genes or the administration of antipsychotic. The reason for homeostasis in schizophrenia ascertains that increased risk of thrombogenesis in schizophrenic patients with antipsychotics treatment has been reported. Second-generation antipsychotics such as Olanzapine, Risperidone and Clozapine are associated with increased dose-dependent risk of venous thromboembolism[95,96] which result from partially inhibition of platelet aggression in schizophrenic patients[97].

### Regulatory pathways of involving under-expressed candidate genes in schizophrenia

**Figure 7:**
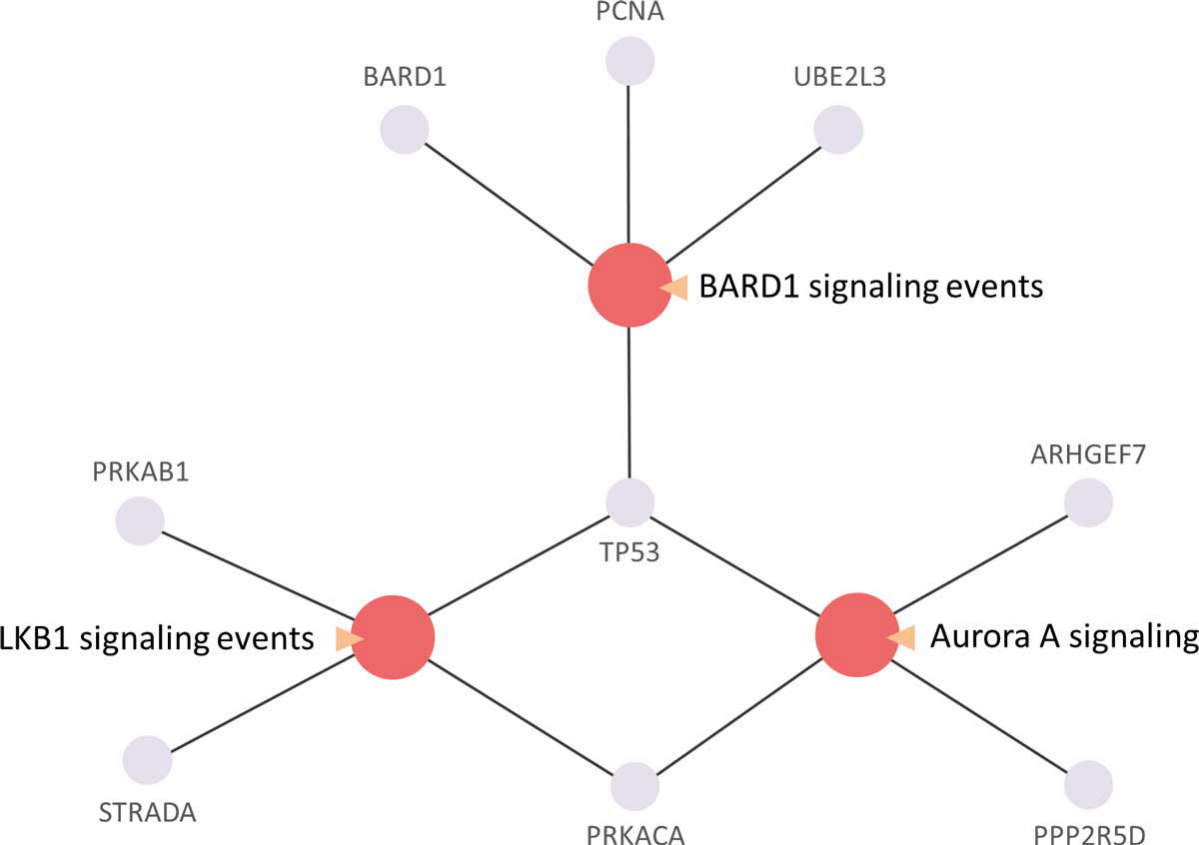
**Under-expressed enrichment pathway network for schizophrenia**. These proteins involved in three signaling pathways including BARD1 signaling events, LKB1 signaling events and Aurora A signaling reveals the under-expressed enrichment pathways which may implicate key roles in pathogenesis of schizophrenia.

In this study, three candidate pathways are associated with under-expressed candidate genes (Figure 7). LKB1 signaling pathway interacts with under-expressed genes PRKAB1 and STRADA. PRKAB1 is associated with polymorphisms in AMP-activated protein kinase (AMPK) and antipsychotic-induced weight gain[98]. In addition, PRKACA contained cluster which involved neurotransmission related functions may be relevant to the pathophysiology of schizophrenia[16]. It also appears in the candidate genes for schizophrenia in SZGene. It is implicated that AKT/GSK3 pathway may contribute to the development of schizophrenia[99]. Glycogen synthase kinase 3-β (GSK3β) has a vital role in many intracellular signaling pathways which involved in gene transcription, cytoskeletal reorganization, energy metabolism, cell cycle regulation, and apoptosis. GSK3β activity also has been associated with schizophrenia[100]. GSK3β polymorphisms might be involved in Parkinson's disorder and schizophrenia risk[101]. The research findings suggest that LKB1 activity induced transcriptional functions including phosporylation of GSK3β and APC binding to microtubules which mediates the microtubule stabilization in the leading process tip[102]. LKB1 signaling pathway is crucial for neuronal migration in the developing neocortex and dysfunction of LKB1 signaling pathway may play a crucial role in neuronal migration and the development of schizophrenia.

BARD1 signaling pathway contains three under-expressed genes, BARD1, PCNA and UBE2L3, in which UBE2L3 polymorphism is associated with schizophrenia[103], and polymorphism study reveals that BARD1 is risk allele for schizophrenia[68]. It indicates that BARD1 signaling pathway dysfunction may represent a novel susceptibility pathway for schizophrenia.

The Aurora A signaling pathway interacts with under-expressed genes including TP53, PRKACA, ARHGEF7 and PPP2R5D. TP53 is considered as a candidate susceptibility gene for schizophrenia which may play a role in the pathogenesis of schizophrenia[104]. PPP2R5D is in co-expression network in Wnt pathway of schizophrenia tissue sample[98]. It is implicated that inhibition of the Aurora A signaling pathway may be a novel susceptibility pathway for schizophrenia.

### Hedgehog signaling pathway and schizophrenia

The Hedgehog signaling pathway is one of the significant signaling pathways involving schizophrenic candidate genes such as SIN3A, CSNK1G3, PRKACA, and SPOP. The crosstalk of PID candidate pathway for schizophrenia between hedgehog signaling pathway and other cancer-related pathways such as LKB1 signaling events, Aurora signaling pathway, ErbB2/ErbB3 signaling pathway and TNF alpha/NFkB signaling pathway. Crosstalk between hedgehog and other signaling pathways such as PI3K/AKT pathway is critical in the development of embryonic cell and cancer therapies[105]. Genetic variation in MED12 which has been implicated in neural development is associated with schizophrenia, and its mutations link with deregulated GLI3-dependent sonic hedgehog signaling pathway[106]. Antipsychotics such as Clozapine, Chlorpromazine and Haloperidol regulate hedgehog signaling pathway through DHCR7 modulation[107]. Hedgehog signaling pathway may represent a pathogenesis of schizophrenia.

Rho signaling pathway is also responsible for schizophrenia[15]. ARHGAP18 is associated with schizophrenia[108]. ARHG (also known as RHOG) family genes are associated with schizophrenia[109] which encodes a member of the Rho family of small GTPases. Rho proteins promote reorganization of the actin cytoskeleton and regulate cell shape, attachment, and motility. In this study, there are schizophrenic candidate gene including RHOG family genes such as under-expressed genes, ARHGEF38, ARHGAP1, ARHGAP36 and ARHGEF7; and over-expressed genes, ARHGEF1.

### Coagulation network in schizophrenia

The use of second-generation antipsychotics (such as Clozapine, Risperidone and Olanzapine) is associated with an increased risk of venous thromboembolism[95,96]. The biological mechanisms responsible for the possible thrombosis reaction are not quite clear. In schizophrenia-coagulation network, the SCZCGs including F8, FN1 and FGL1 are differential expressed candidate genes which are associated with coagulation function. They may account for the variant gene expression by not only schizophrenia itself, but also by antipsychotic administration.

Some hypotheses have been suggested such as drug-induced sedation, obesity, enhanced platelet aggregation, increased levels of antiphospholipid antibodies, hyperprolactinemia, hyperhomocysteinemia and smoking which result in increased activity in the coagulation system[12-14]. However, evidence of venous thromboembolic events in schizophrenia may be induced by psychosis itself rather than by antipsychotic administration[8]. Moreover, chronic anticoagulation therapy (such as warfarin) is associated with remission of psychotic symptoms, which suggest that imbalance of plasminogen activator levels in the brain may affect the stabilization of psychotic symptoms[10].

In this study, SCZCGs are discovered and associated with coagulation function, which may result from the variant gene expression by not only schizophrenia itself, but also by antipsychotic administration. This network also implicated the genetic interactions between hemostasis pathway and antipsychotic associated genes (DRD2, DRD3 and HTR2A) which implicated the potential explanation of increased coagulation activity in antipsychotic treatment.

### The relationship between dopamine, serotonin and coagulation function

The well-understood pathophysiology of schizophrenia is cytoskeleton deficit and cell cycle. Cytoskeleton-associated proteins play crucial roles not only in cell regulation and migration but also in cell proliferation. A cytoskeleton-associated gene mutations in the filamin A (FLNA) cause periventricular heterotopia (PH), cell cycle prolongation, compromised neural progenitor proliferation, and reduced brain size[110]. However, FLNA is one of the novel schizophrenia candidate genes[20] which was involved in the glutamate receptor signaling subnetwork, and might result in brain structure change such as enlargement of the cerebroventricular system, reduced size in cortical region, and abnormal laminar organization with differential genetic changes including abnormal expression of multiple proteins that are involved in cellular functionality such as neuron migration, cell proliferation, axonal outgrowth, and apoptosis[111].

HTR2A interact with DRD3 through GNAI1, DRD2 and DRD3 interact with FLNA cascade the subsequently coagulation associated genes such as F3, F7, F10 and UBC which provides antipsychotic biological mechanism of dopamine D2/D3 receptor subtypes that involved downstream signaling pathway through the cytoskeleton of actin filaments[112,113].

### Signaling pathway of coagulation function in schizophrenia

The risk for cardiovascular mortality among those with schizophrenia is increased twofold as compared with patients without schizophrenia[114]. There is increasing evidence of association between venous thromboembolism and antipsychotic administration[115], especially second-generation antipsychotics including Clozapine, Olanzapine and Risperidone[116,117]. The pathogenesis of elevated risk of thrombosis for schizophrenic patients are not clear, which result from poor life style, enhanced platelet aggregation, sedative medication, obesity, and lack of movement[118]. It is implicated that the risk of VTE in schizophrenia may result from disease itself instead of the antipsychotic medication[13].

**Figure 8:**
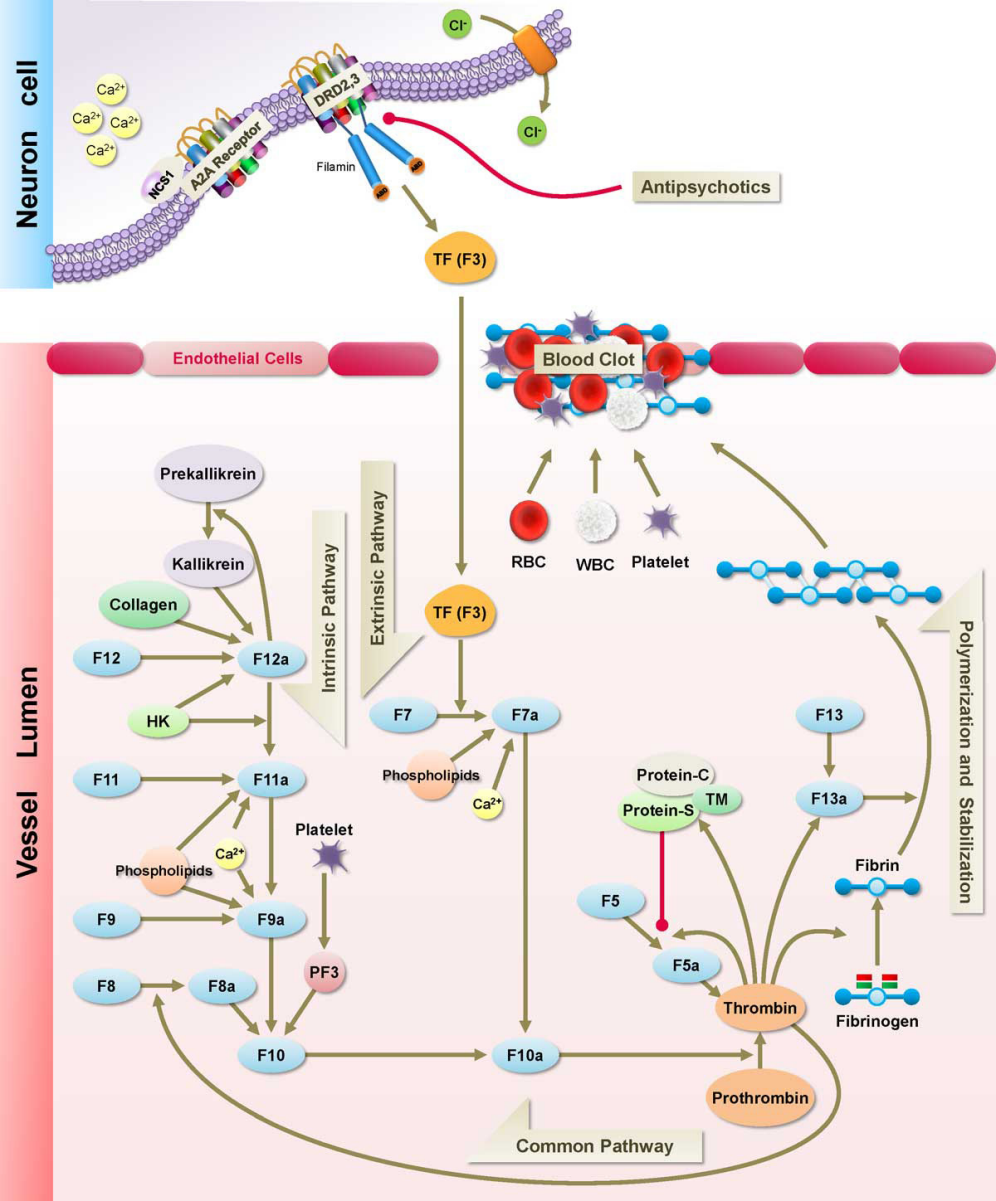
**Signaling pathway of coagulation cascade for schizophrenia**. This figure illustrates the signaling pathway of coagulation process for schizophrenia. The modulation of G-protein couple receptor on the cell membrane involved the existence of A(2A)-NCS-1 complex by neuronal calcium binding protein, NSC-1 and Adenosine A2a receptor. The tissue factor triggered by antipsychotic-associated DRD2/3 receptor was released into vessel lumen to activate the cascade(F3, F7 and F10) of extrinsic pathway of coagulation process. In cluster 39, NCS-1 gene is associated with inhibition of dopamine D2 desensitization, and its expression was decreased in schizophrenic patients. The under-expressed genes in cluster 39 might contribute to the potential pathogenesis of schizophrenia.

Figure 8 illustrated the possible mechanism proposed for the explanation of molecular changes in coagulation pathway in schizophrenia. The stress exposure activated the modulating mechanism of DRD2/3 through cytoskeletal protein interaction of FLNA in neuronal cell[112,119,120], which induce F3 gene expression to the release of tissue factor[121]. The gene-stress model revealed the stress exposure magnitude are associated with DRD2 genotype[122], which induced the expression of TF gene by increased SP1 transcriptional activity[123], which may trigger release of tissue factor to activate the extrinsic coagulation pathway containing FVII/FVIIa induced platelet aggregation and initiate signaling cascade for the extrinsic blood coagulation[124] which cascade the following hemostatic reaction in neuron cell. Moreover, oxidative stress contributes to activate the over-expressed gene of tissue factor in the clotting system[125].

## Conclusions

The big data analysis of NGS by high-throughput technology brought new insights to the global view of complex diseases, which helped to explain the interrelationships among potential pathways and protein complexes, and to discover biological function from the corresponding cortical region. The next generation data of RNA sequence revealed more detailed transcriptional alteration in schizophrenia.

In this study, the comparison and integration of differential expression candidate genes are analyzed from different literatures to explore the relationships between the mitochondrial dysfunction and hemostatic pathways in schizophrenia and control samples. By the integration of transcriptome alteration in NGS BA22 brain tissue and analysis of differential expression candidate genes, the SCZCGs of schizophrenia provide a major approach for the discovery of potential complexes and pathways, which investigation of potential schizophrenic pathology with mitochondrial and coagulation pathways. It is implicated that the hypofunction of mitochondria may contribute to the pathogenesis of negative symptoms of schizophrenia such as hypoactivity, loss of energy, weakness and social withdraw, which restoration of mitochondria function or energy metabolism might be a potential strategy for improvement of the negative symptoms of schizophrenia.

The thromboembolism in schizophrenia revealed the potential alteration of hemostatic pathways which may result from the interaction of antipsychotic target genes with coagulation factor genes. The critical role of HTR2A and DRD2/3 interact with the tissue factor (F3) through FLNA which potentiates a bottleneck in coagulation network. It may illustrate the potential mechanism of thromboembolism in schizophrenia.

In analysis of SCZMN, the clusters screened by the gene function classification tool may reveal the candidate complexes or pathways to discover the potential pathogenesis for schizophrenia. It helped to explain the interrelationships among potential pathways and protein complexes, and to discover biological function from the corresponding cortical region. The potential hemostatic process and mitochondria-related complexes could be implicated for the disease mechanism of schizophrenia.

## Competing interests

The authors declare that they have no competing interests.

## Authors' contributions

KCH interpreted the results, drafted the manuscript, and contributed to the design of the bioinformatics analysis tools. SAL programmed the bioinformatics analysis tools and carried out the data analysis. HL assisted in the interpretation of results. SAL and KCY conceived the study and participated in coordination and management of the research project. TTHT assisted in the drafting and correction of manuscript.

## Supplementary Material

Additional file 1 The released human brain BA22 transcriptome NGS data profile from NCBI SRA database. Click here for file

Additional file 2 The schizophrenic candidate genes compared from different literature reviews. Click here for file

Additional file 3 The expression level of BA22 transcriptome in schizophrenia and control samples with p-value and fold change. Click here for file

Additional file 4 624 enrichment pathways selected from several pathway databases with matched SCZCGs in pathways by their significance in p-value and FDR. Click here for file

Additional file 5 The involved over-and under-expressed genes in MCL clusters with p-value. Click here for file

## Declarations

Publication costs for this article were funded by Kainan University, Taoyuan, Taiwan.

This article has been published as part of *BMC Genomics *Volume 15 Supplement 9, 2014: Thirteenth International Conference on Bioinformatics (InCoB2014): Computational Biology. The full contents of the supplement are available online at http://www.biomedcentral.com/bmcgenomics/supplements/15/S9.
